# Transurethral Incision of the Bladder Neck in a Woman with Primary Bladder Neck Obstruction after Kidney Transplantation

**DOI:** 10.1155/2015/312084

**Published:** 2015-03-16

**Authors:** Dean Markić, Maksim Valenčić, Anton Maričić, Kristian Krpina, Dražen Rahelić, Juraj Ahel, Nino Rubinić, Lidija Orlić, Sanjin Rački

**Affiliations:** ^1^Department of Urology, University Hospital Rijeka, 51000 Rijeka, Croatia; ^2^Department of Nephrology and Dialysis, University Hospital Rijeka, 51000 Rijeka, Croatia

## Abstract

Voiding dysfunction is frequently seen in the early posttransplant period. Among other causes, this condition can arise due to bladder outlet obstruction. Primary bladder neck obstruction (PBNO) is a possible but very rare cause of bladder outlet obstruction. We present the case of a 52-year-old woman who, after kidney transplantation, presented with PBNO. The diagnosis was established based on symptoms, uroflowmetry, and multichannel urodynamics with electromyography. The transurethral incision of the bladder neck was made at the 5- and 7-o'clock position. After the operation, the maximal flow rate was significantly increased, and postvoid residual urine was decreased compared to the preoperative findings. The patient was followed for 5 years, and her voiding improvement is persistent. This is the first reported case of PBNO treated with a transurethral incision of the bladder neck in a posttransplantation female patient.

## 1. Introduction

Kidney transplantation (KT) is the best treatment modality for patients with end-stage renal disease (ESRD). Before transplantation, patients are placed on a renal replacement therapy, haemodialysis in most cases. During this period, the production of urine decreases with time, and the urinary bladder becomes hypotrophic (disused bladder dysfunction). Bladder capacity and compliance decreased with the longer duration of the haemodialysis [1]. After successful transplantation, urine production started again and micturition and bladder function were restored. Nevertheless, some voiding problems may become evident after years of bladder inactivity.

Bladder outlet obstruction (BOO) in women is an infrequent condition compared to men and is found among 2.7–8% of women referred for an evaluation of their lower urinary tract symptoms (LUTS) [2]. BOO in women can be generally divided, according to the cause, into functional or anatomical obstruction (pelvic organ prolapse, uterine tumour, post-anti-incontinence procedure, urethral stricture, and bladder or urethral tumour) [3]. Functional obstruction can be diagnosed only during the act of micturition, as anatomic abnormalities, which can be connected with patients' symptoms, do not exist. One of the functional causes of BOO is primary bladder neck obstruction (PBNO) [3]. PBNO may present with a variety of symptoms, such as a decreased force of the urinary stream, hesitancy, intermittent stream, incomplete emptying, frequency, urgency, urge incontinence, nocturia, and urinary retention [3]. Here, we present a case of a woman after renal transplantation with BOO caused by PBNO who was treated with a transurethral incision of the bladder neck.

## 2. The Case Report

Our female patient has ESRD caused by Alport syndrome. The beginning of her complaints was in childhood. Gradually her kidney function declined and she started with hemodialysis at the age of 25. At the age of 29 she received a kidney transplant from a deceased donor. The first graft lasted 14 years. The cause of graft failure was chronic graft nephropathy and she again started with hemodialysis. At that time the patient was anuric. Three years later the patient had received a kidney transplant (second transplantation) from a deceased donor. There was good match in the human leukocyte antigens (HLAs), with only three mismatches. After transplantation, immunosuppression was started with cyclosporine, mycophenolate mofetil, and prednisolone. Because of gastrointestinal side-effects, mycophenolate mofetil was replaced with azathioprine. The further postoperative clinical course was uneventful, and the function of the transplanted kidney was excellent during follow-up. Also, the patient's bladder function recovery after KT was established in just few weeks.

In the age of 52 our patient came to the urology office due to her frequency, nocturia, decreased force of urinary stream, and feeling of incomplete bladder emptying. The symptoms had started 22 months previously. Her urinary diary showed that she voided 12 times during 24 hours (nocturia 2x) with urine volume of 1500–3000 mL through 24 hours. Laboratory exams showed normal renal function with urea 4.7 mmol/L and creatinine 85 *μ*mol/L. Physical examination and urinalysis were normal. Urinary infection was excluded by sterile culture. Pelvic ultrasound with a full bladder was performed and a possible mass compressing the urethra was excluded. The postvoidal residual urine was 60 mL. Her gynaecological exam was normal. Cystoscopy was performed using a 17 Fr rigid urethrocystoscope and revealed normal endoscopic findings in the bladder and urethra, except for the trabeculation of the urinary bladder.

**Figure 1:**
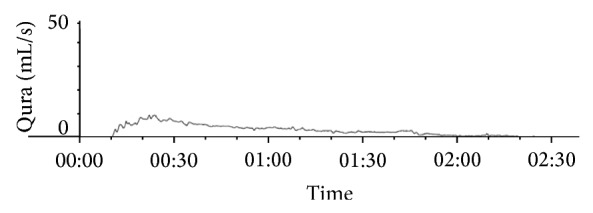
Uroflow before the operation revealed an obstructed flow with a maximal flow rate of 9 mL/s, voided volume of 397 mL, and voiding time of more than 2 minutes.

In our patient, uroflowmetry and multichannel urodynamics were assessed. Uroflowmetry revealed an outflow obstruction, with a maximal flow rate of 9 mL/s (Figure 1). Multichannel urodynamics with electromyography showed normal filling and storage phase, with synchronised activity between the bladder and urinary sphincter during voiding.

The diagnosis of PBNO was established based on the symptoms, uroflowmetry, and multichannel urodynamics. After the PBNO diagnosis, therapy with tamsulosin 0.4 mg once daily for 2 months was started. However, this therapy failed, and a transurethral incision was the next therapeutic step.

**Figure 2:**
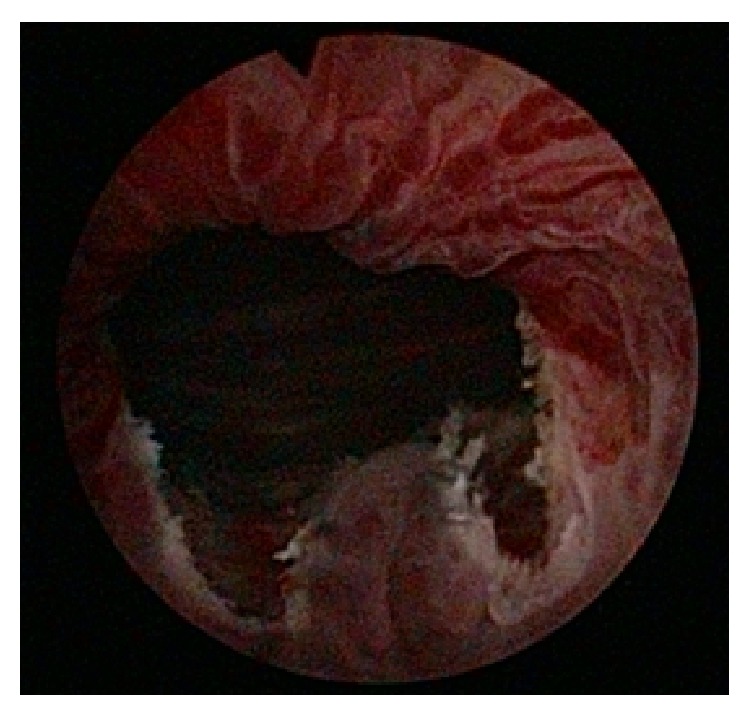
Transurethral incision of the bladder neck in a woman with a primary bladder neck obstruction. Final view of the transurethral incision of the bladder neck at the 5- and 7-o'clock position.

The operation was performed in the standard lithotomy position. The endoscopic incision was made with a vertical diathermy electrode (Collings knife) in the bladder neck at the 5- and 7-o'clock position (Figure 2). At the end of the procedure, the Foley catheter was inserted. The catheter was removed 48 hours after the operation. No procedure-related complications occurred.

**Figure 3:**
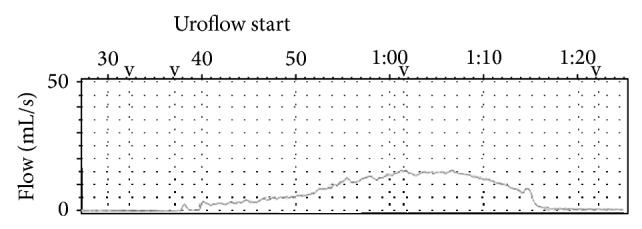
Uroflow after the operation revealed an increased flow with a maximal flow rate of 15 mL/s, voided volume of 351 mL, and voiding time of 45 s.

After the operation, the patient reported a better urinary stream. Her urinary diary showed that she voided 8 times over 24 hours without nocturia. The maximal flow rate was increased from 9 to 15 mL/s, and the postvoid residual urine decreased from 60 to 20 mL compared to preoperative findings (Figure 3). Now, five years after the transurethral incision of the bladder neck, her kidney function is stable, and the patient is still satisfied with voiding, with the improved urodynamics characteristics.

The use of her medical record was approved by the Ethics Committee of the University Hospital Rijeka, and informed consent was obtained.

## 3. Discussion

During the dialysis period, patients with ESRD become oliguric to anuric. Decreased urine production leads to bladder inactivity, followed by atrophic changes in the bladder wall. Bladder capacity and compliance decrease, becoming more pronounced during a prolonged period of dialysis [4]. After KT, the urine production and bladder function are reestablished. Voiding function typically stabilises within 6 months after a successful KT; however, voiding dysfunction and LUTS might persist long-term [5].

The adaptation of the bladder to the newly established production of urine occurs relatively quickly, and urodynamic parameters, such as maximum flow rate, bladder capacity, and bladder compliance, reach their plateaus within 24 weeks after KT [6]. In that period, the voiding function becomes stabilised. Voiding dysfunction, which was assessed 6 months after transplantation by uroflowmetry and postvoid residual urine, was present in 28% of patients. Its prevalence was not related to age, the period of dialysis, time after transplantation, polyuria, or nocturnal polyuria [7]. In addition, approximately 26% of transplanted patients voided more than twice during their sleeping periods [7]. This nocturia may be caused by functional (overactive bladder, PBNO) or anatomical abnormalities (benign prostatic hyperplasia, BPH) but may be secondary to congestive heart failure, diabetes mellitus, hypertension, or lifestyle patterns, such as night-time drinking. Secondary causes can be treated by restricting fluid intake at night, using pharmacological therapies (desmopressin and diuretics) and better blood pressure control [7].

PBNO as a cause of bladder outlet obstruction can be found in 1–16% of obstructed women [3]. PBNO is characterised by the failure of the bladder neck to open adequately during voiding, resulting in weak urine stream. The exact cause of this condition is still unknown [3]. Because anatomical abnormalities are not present, PBNO is considered to be a functional condition. As such, neurological diseases must be excluded as a possible cause in all patients. As the symptoms of patients with PBNO and of those with other functional causes of bladder outlet obstruction are very similar, an exact diagnosis can be made only using multichannel urodynamics with EMG [8].

In patients with PBNO and minor symptoms, watchful waiting may be recommended. If therapy is necessary, alpha blockers are the first-line treatment. The next treatment possibility is a transurethral incision of the bladder neck [8].

The transurethral incision of the bladder neck is the surgical treatment of choice for patients with PBNO. The standard place of a bladder neck incision is at the 5- and 7-o'clock position, with an overall satisfaction rate of 76–100% [3, 8]. The need for a reoperation depends on the number of initial incisions, from 18% in the single incision to 0% in the four incision technique [3, 8, 9]. Possible complications of procedure include urinary incontinence and perforation of the vaginal wall [3, 8, 9]. Injury to the external urethral sphincter is responsible for urinary incontinence and can be seen in up to 6.2% of operated patients [8, 9].

Significant lower urinary tract symptoms after KT can have severe negative effects on patient quality of life, as what occurred in our patient. More importantly, episodes of retention and urinary infections secondary to BPH may pose a risk for renal allograft, up to graft loss [10]. Dion et al. found that LUTS can be improved up to 1 year after transplantation [4]. In male patients, those authors found moderate to severe LUTS in 39% of patients before transplantation, as well as 33% and approximately 20% at 6 and 12 months after transplantation, respectively. Patients with validated questionnaires and pretransplant urine outputs of less than 250 mL per day had the highest risk for moderate to severe LUTS and voiding dysfunction posttransplant. An adequate urological evaluation of these patients, in most cases, is able to show that BPH and overactive bladder are responsible for these symptoms. Medical therapies (alpha blockers, inhibitors of 5 alpha reductase, and anticholinergics) improve symptoms in most patients. Only in a minority of patients surgical therapy is needed.

Disused bladder dysfunction can be expected after KT. Bladder dysfunction will be cleared during early period in posttransplant; otherwise another cause of LUTS should be checked as this case report of PBNO. To the best of our knowledge, this is the first case of a transurethral incision of the bladder neck in women with primary bladder neck obstruction after KT. In the present case, as in the general population, this procedure is minimally invasive and safe. Additionally, high cure rate can be expected after an endoscopic incision of the bladder neck.
